# Parkinson’s Disease is Associated with Dysregulations of a Dopamine-Modulated Gene Network Relevant to Sleep and Affective Neurobehaviors in the Striatum

**DOI:** 10.1038/s41598-019-41248-4

**Published:** 2019-03-18

**Authors:** Peng Jiang, Joseph R. Scarpa, Vance D. Gao, Martha Hotz Vitaterna, Andrew Kasarskis, Fred W. Turek

**Affiliations:** 1 Center for Sleep & Circadian Biology, Department of Neurobiology, Northwestern University, Evanston, IL 60208 USA; 2 Icahn Institute for Genomics and Multiscale Biology, Department of Genetics and Genomic Sciences, Icahn School of Medicine at Mount Sinai, New York, NY 10029 USA; 3 Department of Neurology, Northwestern University Feinberg School of Medicine, Chicago, IL 60611 USA

## Abstract

In addition to the characteristic motor symptoms, Parkinson’s disease (PD) often involves a constellation of sleep and mood symptoms. However, the mechanisms underlying these comorbidities are largely unknown. We have previously reconstructed gene networks in the striatum of a population of (C57BL/6J x A/J) F2 mice and associated the networks to sleep and affective phenotypes, providing a resource for integrated analyses to investigate perturbed sleep and affective functions at the gene network level. Combining this resource with PD-relevant transcriptomic datasets from humans and mice, we identified four networks that showed elevated gene expression in PD patients, including a circadian clock and mitotic network that was altered similarly in mouse models of PD. We then utilized multiple types of omics data from public databases and linked this gene network to postsynaptic dopamine signaling in the striatum, CDK1-modulated transcriptional regulation, and the genetic susceptibility of PD. These findings suggest that dopamine deficiency, a key aspect of PD pathology, perturbs a circadian/mitotic gene network in striatal neurons. Since the normal functions of this network were relevant to sleep and affective behaviors, these findings implicate that dysregulation of functional gene networks may be involved in the emergence of non-motor symptoms in PD. Our analyses present a framework for integrating multi-omics data from diverse sources in mice and humans to reveal insights into comorbid symptoms of complex diseases.

## Introduction

Parkinson’s disease (PD) is a devastating neurodegenerative disorder characterized pathologically by loss of dopaminergic neurons in the substantia nigra pars compacta, reduction of striatal dopamine levels, and aggregation of intracellular protein inclusions, typically containing α-synuclein, termed Lewy bodies. The classical clinical features of PD include resting tremor, rigidity, gait impairment, and bradykinesia, while a range of non-motor symptoms, including sleep dysfunction, mood disorders, cognitive impairment, and dementia, are also often observed^1,2^. Approximately two-thirds of PD patients suffer from some sleep dysfunction^3^, with the most common sleep-related complaints in PD patients being sleep fragmentation (frequent nocturnal awakenings) and excessive daytime sleepiness^4^. PD-related sleep problems also include a range of sleep disorders, particularly rapid eye movement (REM) sleep behavior disorder (RBD), which may represent an early prodromal marker of PD^5^. On the contrary, a night of well-rested sleep may transiently improve motor functions in some PD patients, a phenomenon known as the “sleep benefit”^6^. In addition to sleep disruptions, mild or moderate depressive symptoms are observed in roughly 43% of PD patients^1,7^, and depressive patients show a higher risk of developing PD later in life^8^. These comorbid non-motor symptoms in PD may be associated with the degeneration of sleep and/or mood regulating systems (especially the dopaminergic pathways), adverse effects of chronic medications, and chronic stress^9,10^, although the exact pathophysiological basis is not clear.

Gene expression profiling in various brain regions and genome-wide association studies (GWAS) have identified a number of genes that may be involved in PD pathology. More recently, meta-analyses integrating multiple datasets have been used to minimize the impact of heterogeneity among patient cohorts involved in each individual datasets and produced robust signatures of PD^11–14^. These efforts have associated a number of cellular pathways and processes to PD pathology, including mitochondrial dysfunction, oxidative stress, impaired intracellular calcium homeostasis, autophagy and apoptosis, protein misfolding and proteolytic stress, as well as immune disruptions and inflammation, among others^15,16^. Despite these successes, the mechanisms by which PD-associated genetic and transcriptomic variations lead to a range of motor and non-motor symptoms are not fully understood. Addressing this question requires an understanding of how genes are organized into functional networks underlying motor, sleep, and mood phenotypes and how PD disrupts these gene networks.

Systems biology approaches have been shown effective to describe gene networks that contribute to the emergence of complex physiological functions and pathological conditions, including neurodegenerative disorders^17,18^. We have previously used such an approach to reconstruct gene networks associated with sleep and affective phenotypes in the striatum of chronically stressed (C57BL/6J x A/J) F2 mice^19^, which allow us to interrogate how functional gene networks may be perturbed in diseases, such as the prodromal phase of Huntington’s disease^20^. Here, we report a systems analysis combining these functional gene networks in the mouse striatum with differential gene expression signatures in the striatum of PD patients as well as mouse models, in order to evaluate the functional relevance of PD-associated striatal transcriptomic alterations in the emergence of the motor, sleep, and mood symptoms. We highlight a gene network involved in the regulation of mitotic spindle, circadian clock-controlled gene expression, and Notch signaling. Gene expression in this network was concordantly elevated in the striatum of PD patients and several animal models. In our mouse population, this network was associated with phenotypes that are relevant to the sleep and depressive symptoms observed in PD patients. In our previous analysis of this network using a probabilistic graphical model, the network was regulated by a set of key driver genes that are known for motor functions^19^, thus implying a role of the network in motor symptoms of PD as well. We extensively characterized this striatal gene network using a variety of bioinformatics tools and databases, including cell-type-specific transcriptomic signatures, transcriptomic signatures of genetic and pharmacological perturbations, transcription factor-target databases, and protein-protein-interaction (PPI) databases. We found this network was likely downstream of striatal dopamine receptors and was regulated by a network of CDK1-phosphorylated transcription factors including a GWAS candidate of PD, NUCKS1. These findings suggest striatal dopamine deficiency leads to disruptions in the gene networks associated with motor, sleep, and affective functions, and dysfunctions of these gene networks may ultimately contribute to the spectrum of PD symptoms.

## Results

### A robust differential gene expression signature in the striatum of PD patients

**Figure 1:**
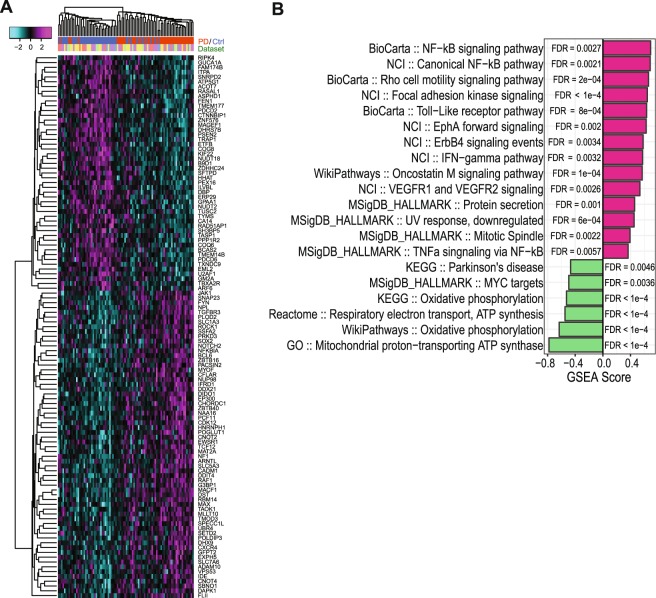
Meta-analysis of differential gene expression in the striatum of PD patients. (**A**) Heatmap of the expression profile of meta-DE genes (FDR < 0.05). Expression values of each gene are standardized within each dataset. Hierarchical clustering was used to cluster samples (columns) and genes (rows). (**B**) Enrichment of gene sets or pathways in up- (positive scores) or down- (negative scores) regulated genes of the PD meta-signature. Top 20 significant gene sets are shown, and the full results are included in Supplementary Table S1.

PD is a heterogeneous disease, and differential gene expression signatures established from transcriptomic profiling studies are typically challenged by large variations amongst different patient cohorts^11,21^, due to factors including the duration and clinical stage of the disease and the treatment history of the patient. Meta-analyses combining information from multiple studies have been demonstrated to successfully combat this challenge to capture the core pathophysiology^22^, as exemplified by a previous study that combined microarray datasets collected from several brain regions and peripheral blood of PD patients, revealing consistent changes in genes involved in the mitochondrial electron chain^11^. In order to establish a robust PD transcriptomic signature specifically in the striatum, we performed a meta-analysis using five publicly available transcriptomic datasets collected from postmortem striatal samples of PD patients and control subjects (N = 4–15 per group per dataset; Supplementary Table S1). The combined dataset of our meta-analysis contains 10,098 genes that are included in all five datasets. We combined the effect size statistic (Hedges’ g) across datasets for each gene to identify genes that are differentially expressed in PD patients compared to healthy controls^23^. At a false discovery rate (FDR) < 0.05, we identified 64 upregulated genes and 49 downregulated genes in the striatum of PD patients. We then performed hierarchical clustering on the within-dataset-standardized expression data of these 113 genes across all samples, which showed that the differential expression pattern was driven primarily by disease status across all datasets and not biased by any particular dataset (Fig. 1A). We further tested the robustness of our meta-analysis signature and found that the meta-signature was in good agreement with previously reported sets of differentially expressed genes, even those reported by a study not included in our meta-analysis due to lack of raw expression data (Supplementary Fig. S1).

In order to understand the biological implications of the disrupted striatal transcriptome in PD, we used Gene Set Enrichment Analysis (GSEA)^24^ to search for enriched gene sets of biological functions and pathways. As expected, many of the gene groups and pathways highlighted by the meta-signature have been previously implicated in PD striatal pathology (Fig. 1B; Supplementary Table S1). In particular, a decreased expression of genes involved in oxidative phosphorylation and respiratory electron transport was found as the most significantly affected pathways in PD striatum, consistent with the previous meta-analysis across multiple brain regions and peripheral blood^11^. In addition, expression of genes involved in the immune function and inflammation, including NF-κB pathway, IFN-γ pathway, Oncostatin M pathway, and Toll-like receptor pathway, was upregulated in the PD striatum, consistent with the widely reported neuroinflammation in PD and other neurodegenerative diseases^25^. In addition to these pathways commonly highlighted by previous transcriptomic studies, our analysis revealed elevated expression of genes involved in mitotic spindle, Rho-mediated cell migration, focal adhesion, EphA signaling, ErbB4 signaling, and VEGFR signaling. These pathways play important roles in cell proliferation, differentiation, and mobility, dysregulation of which are linked to various cancers. Thus, our observations further support the increasingly appreciated epidemiological and mechanistic links between PD and cancer^26,27^. Taken together, our meta-analysis established a robust transcriptomic signature in the striatum of PD patients, highlighting biological pathways pivotal to PD pathology.

### PD alters sleep and affective gene networks in the striatum

In addition to dissecting the meta-signatures into cellular and molecular pathways, we investigated how PD-associated transcriptomic changes in the striatum influence gene networks that are associated with PD-relevant sleep and affective functions. We have previously reconstructed gene coexpression networks in mouse striatum using RNA-Seq data obtained from 100 chronically stressed (C57BL/6J x A/J) F2 mice in which we measured 15 categories of 328 sleep and affective phenotypes^19^. We have reported the identification of 62 coexpressed network modules, which were named by randomly assigned colors. We determined the functional relevance of these network modules to sleep and affective functions by computing correlations between module eigengenes (i.e., the first principal component) and phenotypes in each phenotypic category. In the present study, we combined these functionally characterized gene networks in mice with the meta-signature established in PD patients, in order to investigate the biological significance of PD-associated transcriptomic disruptions in the striatum, especially with regard to the emergence of comorbid sleep and mood symptoms in PD.

**Figure 2:**
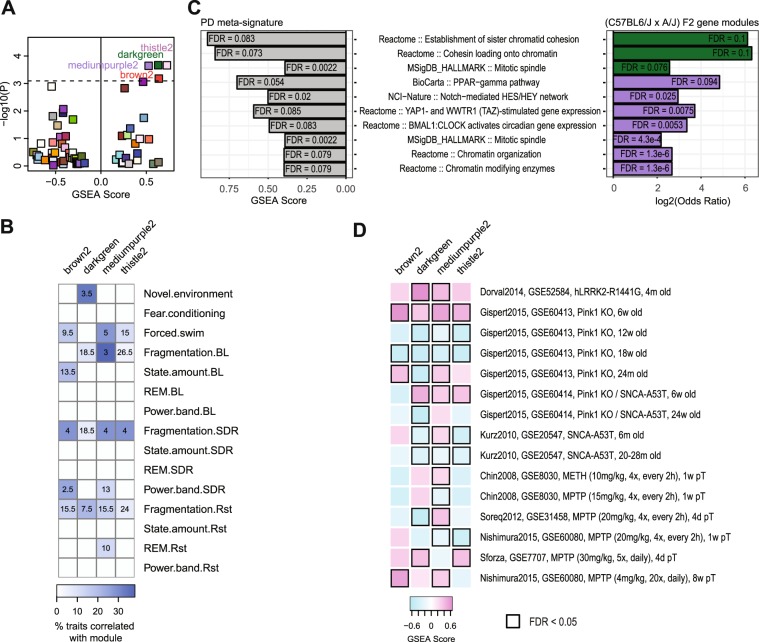
Mouse striatal gene networks differentially expressed in PD. (**A**) Enrichment of mouse gene networks in the PD differential expression signature, as an indication of network-level differential gene expression. Each square represents a network module, and modules significantly enriched in PD signatures are noted with module names. (**B**) Categories of sleep and affective phenotypes correlated with PD-associated modules. Each row represents a category of sleep or affective behavioral phenotypes measured in the (C57BL6/J x A/J) F2 mouse population, and each column represents a gene coexpression network module identified using this dataset. Heatmap color represents the percentage of phenotypes in each category that are significantly (P < 0.05; FDR < 0.05) associated with the module, re-plotted according to results from our previous study^19^. Categories of affective behavioral phenotypes included activity levels and anxiety-like behaviors when exposed to a novel environment (such as those measured in the open field, elevated plus maze, and elevated zero maze), behavioral responses measured in the fear conditioning test, as well as activity levels and despair-like behaviors measured during the forced swim test. Sleep phenotypes included those measured under 24 hours of undisrupted baseline condition (BL), changes in sleep/wake during the recovery after a 6-hour sleep deprivation compared to the equivalent time period during baseline (SDR) and sleep/wake changes after a 15-minute restraint stress compared to the equivalent time period during baseline (Rst). Sleep phenotypes under each conditions were grouped into categories including sleep fragmentation (such as the duration or numbers of sleep episodes and the frequency of changes in sleep/wake states), state amount (the amount of sleep or wake during a given time interval), rapid-eye-movement (REM) sleep measurements, and intensity (i.e. power) of EEG frequency bands. For each category, modules were ranked based on the number of significant phenotypic associations in that category, and the text in each cell indicates the rank of the module among all 62 modules^19^. An averaged rank was used when modules tied for the relevance to a category. (**C**) Gene sets enriched in both PD meta-analysis signature (left) and PD-associated modules (right). Only gene sets enriched (FDR ≤ 0.1) with up-regulated genes (positive GSEA scores) are included, since all modules highlighted by the meta-signature were enriched with up-regulated genes. Two of the PD-associated modules (Darkgreen and Mediumpurple2) shared enrichment of pathway and cellular functions with the PD meta-signature, and the bar plot (right) for enriched modules was colored according to the module names. (**D**) Heatmap showing the enrichment scores of PD-associated gene networks (columns) in differentially expression signatures established using various PD models in mice (rows), labeled by the author, year, data accession, and model descriptions. Abbreviations: KO, knockout; METH: methamphetamine; MPTP: 1-methyl-4-phenyl-1,2,3,6-tetrahydropyridine; pT: post-treatment.

We then used GSEA to summarize gene-level differential expression to the network level for each of the network modules. In this analysis, a significant GSEA score indicates that the overall distribution of gene-level differential expression in the network was shifted toward increased expression (i.e, enriched with upregulated genes; a positive score) or decreased expression (i.e., enriched with downregulated genes; a negative score), and thus suggests a network-level change in gene expression caused by PD. We identified four network modules that were enriched with genes upregulated in PD, suggesting that PD striatal pathology altered the transcriptome at the network level (Fig. 2A). Our previous study has linked these PD-associated gene networks to a range of sleep and affective phenotypes (Fig. 2B)^19^. In particular, the Mediumpurple2 module was strongly associated with sleep fragmentation phenotypes (such as the number and duration of sleep/wake bouts) measured under undisturbed baseline conditions. This observation suggests that fragmented sleep, the most common sleep complaint in PD, may be linked to altered expression of striatal gene networks that are particularly relevant to the stability of sleep/wake state. In addition, two of these PD-associated network modules were also highly relevant to behavioral despair phenotypes measured in the forced swim test (Mediumpurple2) and anxiety-like behaviors when exposed to a novel environment (Darkgreen)^19^, recapitulating comorbid mood symptoms commonly reported in PD patients. Together, these findings link gene expression changes in PD to gene networks associated with phenotypes that are relevant to the non-motor symptoms of PD.

While our pathway analysis of the meta-signature identified striatal cellular functions disturbed by PD (Fig. 1B), it does not explicitly link to PD symptoms. To address this, we reasoned that cellular functions highlighted by both the meta-signature and the PD-relevant sleep/affective networks are particularly relevant to the emergence of PD sleep and mood symptoms. We thus examined the molecular and cellular functions of PD-associated sleep/affective gene networks and identified the functions whose gene expression were also changed in the same direction in the striatum of PD patients (Fig. 2C). In addition to mitosis-/cancer-related pathways (mitotic spindle and YAP1 and WWTR1 controlled genes expression), molecular functions highlighted by this analysis included chromatin modification and organization, PPAR-gamma pathway, Notch signaling, circadian clock regulated gene expression, and mRNA processing, suggesting potential molecular processes linking striatal pathology of PD to the emergence of sleep and mood symptoms.

We next investigated whether the gene expression levels in these PD-perturbed sleep/affective gene networks were altered also in the striatum of PD mouse models. We collected striatal transcriptomic datasets from 7 studies of various genetic and neurotoxin models of PD in mice, and we tested if the PD-associated gene networks could be validated in each of these datasets. The patterns of network-level differential gene expression were highly variable in different mouse models of PD, perhaps due to the differences in the neuropathology and behavioral phenotypes in these models. Even within the same PD model, for example, *Pink1* knockout mice, differential gene expression at the network level can be in opposite directions at different ages, which may be related to striatal compensatory mechanisms in response to PD pathology and/or variations in cell-type proportions at different stages of the disease. Despite the heterogeneity of PD mouse models, we are particularly interested in network modules whose gene expression was elevated across multiple mouse models, similar as in PD patients, because they are likely associated with critical aspects of PD striatal pathology that are commonly captured by many of the PD mouse models. Significantly increased network gene expression was observed in 8 datasets for the Mediumpurple2 module, 4 datasets for the Darkgreen module, and 3 datasets for the Brown2 and Thistle2 modules (Fig. 2D). The Mediumpurple2 module appeared to be the most robustly upregulated network across a range of genetic and neurotoxin models of PD, including the *LRRK2-R1441G* transgenic mice, *Pink1* knockout mice (6 and 24 weeks old), *SNCA-A53T* transgenic mice (6 months old), *Pink1*^−/−^/*SNCA-A53T* double mutant mice (6 weeks old), methamphetamine-treated mice, as well as two MPTP (neurotoxin 1-methyl-4-phenyl-1,2,3,6-tetrahydropyridine) models. Taken together, expression of the Mediumpurple2 module was elevated in the striatum of PD patients as well as a number of PD animal models, and the network was strongly associated in a large mouse population with sleep and despair behaviors relevant to the symptomatology of PD. Therefore, we identify the Mediumpurple2 network as a candidate molecular correlate of sleep and affective functions relevant to the non-motor symptoms observed in PD.

### The Mediumpurple2 network was regulated by dopamine signaling

**Figure 3:**
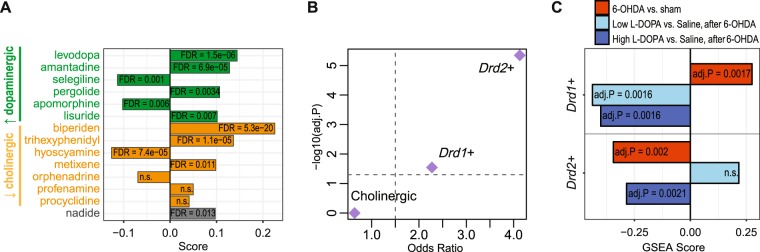
Mediumpurple2 is a striatal dopamine-responsive network. (**A**) Concordant up- (positive scores) or down- (negative scores) regulation of the Mediumpurple2 module genes induced by 11 out of 14 PD drugs included in the CMap data. Drugs that enhance dopaminergic or inhibit cholinergic neurotransmissions are highlighted in green or yellow, respectively. (**B**) Enrichment of the Mediumpurple2 module genes in cell-type-specific signatures of mouse striatal neurons (dashed line: odds ratio = 1.5, Bonferroni adjusted P = 0.05). (**C**) Enrichment of the Mediumpurple2 module genes in cell-type-specific differential expression signatures induced by PD-like insult and chronic levodopa treatment *in vivo*. P values were adjusted using Bonferroni correction.

Since we identified mouse striatal gene networks that were differentially expressed in PD and associated with PD-relevant sleep/affective behaviors, we reasoned small bioactive molecules that perturb these networks may be used to ameliorate Parkinsonian symptoms. We examined this hypothesis by comparing the PD-associated network modules to over 6,000 transcriptomic profiles collected by the Connectivity Map (CMap) project in which the transcriptomic effects of 1,309 compounds were tested on a number of human cancer cell lines^28^. Interestingly, we found that the Mediumpurple2 module was enriched with the signatures of 11 out of the14 drugs that treat PD symptoms (Fig. 3A). Most of these drugs either enhance dopaminergic signaling or suppress cholinergic signaling, and treat PD symptoms via restoring the balance of dopaminergic and cholinergic activities in the striatum. We thus hypothesized that the Mediumpurple2 module may be downstream of dopaminergic or cholinergic neurotransmission. To test this, we compared the PD associated modules to the striatal cell-type signatures produced by translating ribosome affinity purification (TRAP)^29^. We observed an enrichment of the Mediumpurple2 genes in the cell-type specific signatures of medium spiny neurons (MSNs) that express dopamine receptor gene *Drd1* or *Drd2* (i.e., *Drd1*+ or *Drd2*+ MSNs), but not cholinergic neurons (Fig. 3B). We further examined cell-type-specific signatures of differential gene expression in a mouse 6-hydroxydopamine (6-OHDA) model of PD as well as the responses to chronic treatment with a high or low dose of chronic levodopa in this mouse model^30^. We found that the expression of Mediumpurple2 module genes in the striatal *Drd1*+ MSNs was significantly elevated in the 6-OHDA-lesioned mice, and this increased expression was reversed when the lesioned animals were chronically treated with levodopa at either a high or a low dose (Fig. 3C). In the *Drd2*+ neurons, however, the expression of Mediumpurple2 network genes was decreased in the 6-OHDA-lesioned mice and was further decreased when the mice were treated chronically with a high dose of levodopa (Fig. 3C). These results suggest that the Mediumpurple2 network is downstream of striatal dopamine receptors and is sensitive to the treatment of dopaminergic agents, although the dopaminergic signaling cascades that regulate this network in the striatal *Drd1*+ and *Drd2*+ MSNs may be differentially perturbed by PD-like pathology.

### CDK1 is a likely network regulator of the Mediumpurple2 module

**Figure 4:**
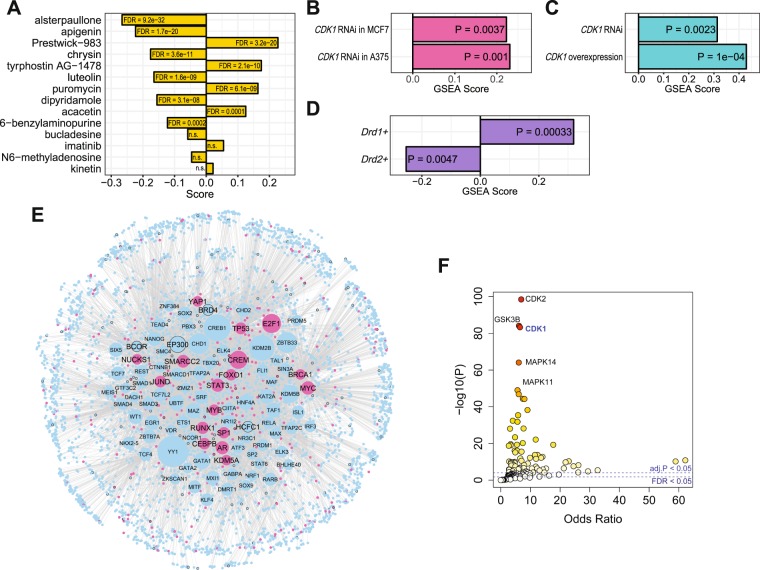
CDK1 is a likely regulator of Mediumpurple2. (**A**) Concordant up- (positive scores) or down- (negative scores) regulation of the Mediumpurple2 module genes induced by drugs known or predicted to target CDK1. (**B**) Concordant up-regulation (positive scores) the Mediumpurple2 module genes induced by knockdown of *CDK1* in A375 and MCF7 cells. (**C**) Mediumpurple2 genes were upregulated by both RNAi knockdown and overexpression of *CDK1*, according to consensus signatures of *CDK1* perturbation aggregated across multiple human cancer cell lines. (**D**) Mediumpurple2 was enriched for genes positively correlated with *CDK1* expression in *Drd1*+ medium spiny neurons, but was enriched for genes negatively correlated with *CDK1* in *Drd2*+ neurons. (**E**) Striatal TF PPI network of the Mediumpurple2 module. Larger nodes denote TFs whose targets are significantly enriched in the module, and the node size is proportional to the –logP value. Pink nodes indicate phosphorylation targets of CDK1. Nodes with a black rim indicate genes coexpressed in the Mediumpurple2 module. (**F**) Enrichment of phosphorylation targets of kinases in the Striatal TF PPI network depicted in (**E**). Data points are colored according to the P value. Dashed lines indicate P-value thresholds at FDR < 0.05 and Bonferroni corrected P < 0.05.

In order to find additional classes of compounds that coherently induce transcriptomic changes perturbing the Mediumpurple2 module, we took advantage of a previous effort that systematically annotated known and predicted targets of each compound used in the CMap project^31^, and computed an enrichment score (a Kolmogorov–Smirnov-like statistic) for each of the target proteins. Interestingly, the top enriched target protein is CDK1 (score = 0.48, FDR = 0.015), a cell cycle regulator known to modulate a number of Mediumpurple2 network functions such as mitotic spindle^32^. 10 out of the 14 CDK1-perturbing compounds in CMap produce differential expression signatures that are enriched with the Mediunpurple2 module genes (Fig. 4A). It is important to note that these compounds do not specifically target CDK1, but instead induce transcriptional changes that previous studies have associated with CDK1 regulation. The finding of CDK1 as a top enriched target suggests that perturbing CDK1 is a common feature of a group of Mediumpurple2-modulating compounds and thus CDK1 is a strong candidate as a regulator of the network. In order to verify this finding, we studied publicly available data measuring transcriptomic effects of perturbing CDK1, obtained from two independent microarray experiments (GSE31534 and GSE31912) in an RNAi screen study that profiled gene expression in response to *CDK1* knockdown in A375 (a human melanoma skin cell line) and MCF7 (a human breast cancer cell line)^33^. Remarkably, *CDK1* knockdown in both datasets induced an increased expression of Mediumpurple2 genes (Fig. 4B). These changes are consistent in direction with the observations from our meta-analysis, in which *CDK1* expression was moderately decreased in the striatum of PD patients (Z-score = −2.74, FDR = 0.103; Supplementary Table S1) while the expression of Mediumpurple2 module genes was increased. We further verified this observation using data from the NIH LINCS program^34^, which is a significant scale-up of the CMap project and investigates transcriptomic signatures of genetic and pharmacological perturbations in 9 human cancer cell lines of different tissue origins. Unlike the CMap project, transcriptomic signatures from LINCS are computationally inferred from 978 measured landmark transcripts. We derived consensus signatures of *CDK1* RNAi or overexpression across all cell lines, again revealing altered expression of Mediumpurple2 network genes in response to *CDK1* perturbations, although knockdown and overexpression of *CDK1* both elevated the expression of Mediumpurple2 module genes (Fig. 4C). Therefore, despite the complexity in the direction, gene expression in the Mediumpurple2 module was modulated at the network level by genetic and pharmacological perturbations of CDK1, suggesting that CDK1 is a candidate network regulator of Mediumpurple2.

Since the CDK1 perturbation datasets in CMap and LINCS involves only non-neuron cell lines of various tissue origins, we next hypothesized that CDK1 also regulates the Mediumpurple2 network in striatal neurons, particularly *Drd1*+ and *Drd2*+ MSNs. To test this hypothesis, we reasoned that, if such a regulatory relationship exists, the expression levels of Mediumpurple2 genes might be among those most correlated with *CDK1* expression in the striatal *Drd1*+ and *Drd2*+ MSNs, especially when under PD-like insult and dopamine treatment. We utilized the cell-type specific transcriptomic dataset from the striatal *Drd1*+ and *Drd2*+ MSNs of 6-OHDA- and levodopa- treated mice^30^, and computed correlations between *CDK1* and all other genes in the genome to form a CDK1-association signature. We then applied GSEA to test whether the Mediumpurple2 network was enriched with genes positively or negatively correlated with *CDK1*. This analysis revealed further the complex relationship between *CDK1* and Mediumpurple2 gene expression. The Mediumpurple2 module was enriched with genes positively correlated with *CDK1* in *Drd1*+ MSNs, but in *Drd2*+ MSNs, it was enriched with genes negatively correlated with *CDK1* (Fig. 4D). This observation confirms a relationship between the expression of *CDK1* and the expression of Mediumpurple2 module genes in the striatal *Drd1*+ and *Drd2*+ MSNs, although the direction of the relationship appeared to be cell-type specific.

### CDK1 regulates the Mediumpurple2 module via a transcription factor protein network

The cellular functions of CDK1, particularly in the regulation of mitosis, involve coordinating the transcriptional activities of a network of transcription factors (TFs) by phosphorylating these TFs^35^. We thus hypothesized that the potential mechanisms by which CDK1 influences the network-level gene expression in the Mediumpurple2 module may involve phosphorylating TFs that regulate large proportions of the Mediumpurple2 genes. In order to test this hypothesis, we first searched the ChEA database^36^ and the ENCODE project data portal^37^ for TF-target relationships established by ChIP-seq or ChIP-on-chip (i.e., chromatin immunoprecipitation followed by sequencing or microarray) studies. We tested 403 TFs for the enrichment of their transcriptional targets in the Mediumpurple2 module identified 100 TFs with significantly enriched targets at FDR < 0.01. Among these Mediumpurple2-regulating TFs, we found a moderate but statistically significant overrepresentation of those phosphorylated by CDK1 (17 TFs; odds ratio = 1.93, P = 0.036; Fig. 4E), according to the kinase-substrate relationships hosted by the KEA database^38^.

Since the regulation of Mediumpurple2 network gene expression by CDK1 may also involve phosphorylation of TF binding factors and regulatory proteins in addition to the TFs themselves, we constructed a protein-protein interaction (PPI) network around the Mediumpurple2-regulating TFs to include their interacting proteins. We then searched the TF-PPI network of the Mediumpurple2 module for known substrates of kinases categorized by the KEA database. We found that CDK1 ranked 3^rd^ for the enrichment of its phosphorylation targets in the TF-PPI network (Fig. 4E,F), providing orthogonal evidence that CDK1 is a candidate regulator of the striatal dopamine-responsive network Mediumpurple2. Other top kinases, such as MAP kinases and GSK3B, have also been implicated in Parkinsonian pathology or levodopa-induced dyskinesia via modulation of postsynaptic dopamine signaling in the striatal MSNs^39,40^. In addition, a remarkable proportion (41.8%) of the Mediumpurple2 module can be found in its TF-PPI regulatory network (odds ratio = 2.39, P = 4.15 × 10^−13^), suggesting the Mediumpurple2 network may be involved in controlling the coexpression of its own genes via transcriptional regulatory protein networks. Taken together, our findings suggest potential mechanisms underlying the transcriptional regulation of Mediumpurple2 network, and suggests CDK1 is likely a regulator of this PD-relevant striatal gene network.

### PD genetic susceptibility highlights a CDK1-NUCKS1 regulatory pathway upstream of the Mediumpurple2 network

**Figure 5:**
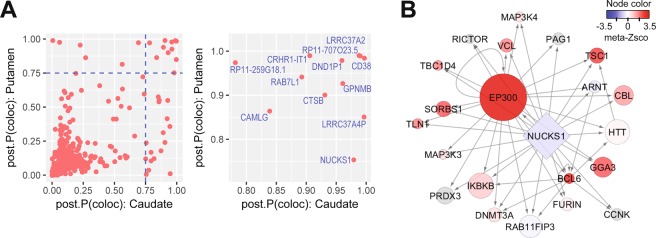
Mediumpurple2 network is regulated by a PD GWAS candidate gene NUCKS1. (**A**) LEFT: Posterior probabilities of colocalization between an eQTL and a PD GWAS locus. Each circle represents a gene whose eQTL in the putamen and caudate was compared to PD GWAS signals. Dashed lines: significant threshold of posterior probability = 0.75. RIGHT: Enlarged upper-right corner of the LEFT, showing significant genes with a posterior probability >0.75 in both striatal regions. (**B**) Direct transcriptional targets of NUCKS1 in the Mediumpurple2 module. Each node denotes a gene and each directed edge denotes ChIP-determined transcriptional regulatory relationships according to data from the ChEA and ENCODE databases. Node Colors indicate the meta-Z-statistic of differential gene expression from the meta-analysis of PD striatal transcriptomic datasets. Genes not included in the meta-analysis were represented by light grey nodes. Round nodes denote genes in the Mediumpurple2 module, and their sizes are proportional to the within-module connectivity computed in WGCNA of our previous work^19^.

In order to test whether the Mediumpurple2 network is influenced by PD genetic susceptibility, we studied striatal transcriptional variations that are regulated by genetic susceptibility loci of PD. We queried the PDGene database^41^, which hosts the summary statistics of the top 10,000 SNPs identified from the largest PD meta-GWAS to date^12^, and we combined this dataset with the expression quantitative trait loci (eQTL) data in two striatal structures, putamen and caudate, from healthy subjects studied by the Genotype-Tissue Expression (GTEx) project^42^. We used a Bayesian colocalization test^43^ to identify genes whose expression in the putamen or caudate are likely regulated by the PD susceptibility loci. At a posterior probability >0.75, we identified 18 genes in the putamen and 30 genes in the caudate nucleus whose regulating eQTL were colocalized with PD meta-GWAS signals. These included 12 genes that were found in both regions, representing a high-confidence set of striatal genes controlled by PD genetic susceptibility (i.e., PD GWAS-eQTL genes; Fig. 5A).

Although PD GWAS-eQTL genes were not enriched in the Mediumpurple2 module or its TF-PPI regulatory network, three of them (*CTSB*, *GPNMB*, and *NUCKS1*) were found in the TF-PPI network. Interestingly, NUCKS1 (nuclear casein kinase and cyclin-dependent kinase substrate 1) is a TF whose transcriptional targets were enriched in the Mediumpurple2 module, and it is a phosphorylation substrate of CDK1 (Fig. 4D). 17 transcriptional target genes of NUCKS1 were coexpressed in the Mediumpurple2 module (Fig. 5B), including *EP300*, a network hub of the module whose expression was significantly increased in the striatum of PD patients (Z-score = 3.52, FDR = 0.029; Supplementary Table S1). *EP300* encodes a TF whose targets were also enriched in the Mediumpurple2 module (Supplementary Table S2), consistent with the role of a top network hub that may relay the regulatory signals from CDK1-NUCKS1 to control the expression of Mediumpurple2 network genes. Taken together, our analysis suggests a CDK1 substrate, NUCKS1, as a nexus linking PD genetic susceptibility to a striatal gene network that is implicated in the emergence of PD symptoms.

## Discussion

PD pathology involves profound transcriptomic disturbances in multiple brain regions, particularly the striatum, which is directly affected by the dopamine depletion in PD and is key to the emergence of PD symptoms. In this study, we performed an integrated analysis combining PD transcriptomic signatures in humans with mouse gene networks that are functionally linked to sleep and affective phenotypes. Unlike previous transcriptomic studies that focused only on gene-level expression and cellular pathways altered in PD, our analysis revealed how high-level functions such as sleep and affective behaviors may be symptomatically altered in PD via network-level concordant differential gene expression. By establishing a robust gene differential expression signature from five publicly available PD transcriptomic datasets, we found that four network modules showed elevated gene expression at the network level in the striatum of PD patients. We particularly a highlighted network module, named Mediumpurple2, as the elevated expression of Mediumpurple2 genes was validated in a range of PD mouse models, despite the heterogeneity in the neuropathology, phenotypes, and disease stages associated with these models. Changes in Mediumpurple2 gene expression may thus be associated with aspects of the striatal pathology or symptoms that are key in PD and commonly represented by multiple PD mouse models, while gene expression changes in the other three PD-associated modules (i.e., Brown2, Darkgreen, and Thistle2) may be related to those captured by only a few mouse models. Indeed, we found the Mediumpurple2 network was related to striatal dopamine signaling, a deficiency in which is the hallmark of PD striatal pathology that is captured by most, if not all, of the PD mouse models. Mediumpurple2 was associated with sleep fragmentation and activities during forced swim in mice. Despite differences in sleep architecture between mice and humans (e.g., mice are nocturnal and sleep in short bouts), the neural and genetic regulatory mechanisms of sleep are relatively conserved between the species. In addition, the associations with sleep fragmentation and behavioral phenotypes during forced swim are only relevant to the normal, non-pathological functions of the Mediumpurple2 network, as the phenotypes were measured in young, healthy mice. Nevertheless, the altered expression of Mediunpurple2 genes in PD implicates a plausible basis for the sleep and mood disruptions seen in PD patients. Furthermore, our previous work has identified network drivers of Mediumpurple2 in a probabilistic graphical model, and 37% of the drivers are known to affect motor functions when mutated^19^. Thus, our findings suggest that motor and non-motor symptoms characteristic of PD may involve shared signaling cascades.

We showed that the Mediumpurple2 network was downstream of nigrostriatal dopamine signaling, as it was enriched specifically with transcriptomic signatures of striatal *Drd1*+ and *Drd2*+ MSNs, and its expression was influenced by PD-like insult and levodopa treatment in those neurons. Both D1 and D2 dopamine receptors are involved in motor control and have been implicated in PD-associated sleep and mood disruptions^44–48^. In the classic direct/indirect pathway model of basal ganglia motor control, nigrostriatal dopamine activates *Drd1*+ MSNs and promotes motor activity (i.e., direct pathway), while dopamine also inhibits *Drd2*+ MSNs, lifting the suppression of motor activity (i.e., indirect pathway)^49^. Interestingly, our analysis showed that the expression of Mediumpurple2 genes was increased in *Drd1*+ MSNs and decreased in *Drd2*+ MSNs in a 6-OHDA model of PD. The distinct changes of Mediumpurple2 expression in *Drd1*+ and *Drd2*+ MSNs in response to 6-OHDA-induced nigrostriatal dopamine depletion may be associated with the opposite downstream actions of striatal D1 and D2 dopamine receptors. When chronically treated with levodopa, Mediumpurple2 gene expression was decreased in *Drd1*+ MSNs, opposite to the change caused by dopamine depletion, which is consistent with the expected treatment outcome of a dopaminergic agent. However, Mediumpurple2 expression was further decreased in *Drd2*+ MSNs upon chronic treatment with a high dose of levodopa, suggesting a complex regulatory mechanism and perhaps a modulated downstream action of D2 receptors by the chronic treatment.

While typical gene-level analysis reveals insights into the cellular pathways and molecular functions disturbed in diseases, it does not necessarily identify which of the disease-affected pathways are particularly important to the symptoms of the disease. Our analysis tied PD-affected cellular pathways to sleep and mood symptoms by matching the enriched pathways of the gene-level differential expression signature to those associated with PD-modulated sleep/affective gene networks. For example, genes involved in circadian clock-regulated rhythmic gene expression and the Notch signaling cascade were enriched both in the PD-upregulated genes and in the Mediumpurple2 network module. The circadian clock plays an important role in the regulation of sleep and mood^50,51^. Disruptions of circadian rhythms have been reported in PD patients^52,53^, and the rhythmic expression of *Per2*, a circadian clock gene, in rat dorsal striatum requires daily activation of D2 dopamine receptors and is blunted by neurotoxin-induced dopamine depletion^54^. Disrupted circadian gene expression in response to dopamine deficiency may be directly relevant to the fragmented sleep in PD, since a reduction in the amplitudes of circadian oscillations, such as the blunted melatonin rhythms in PD patients^53^, could contribute to the disrupted nighttime sleep and excessive daytime sleepiness. Similarly, Notch signaling has been implicated in the regulation of striatal responsiveness to dopamine, particularly involving D1 dopamine receptors^55^, and it regulates sleep-like quiescence in *C*. *elegans* and sleep homeostasis in *Drosophila*^56,57^. Thus, our findings provide insights into specific cellular processes that are key to the emergence of PD non-motor symptoms.

Another important set of genes that are enriched with both the PD-upregulated genes and the Mediumpurple2 network include those involved in mitosis and cancer. This link between PD, Mediumpurple2 network, and mitotic genes was further supported by the finding that a key mitotic checkpoint gene, *CDK1*, is likely to be a network regulator of Mediumpurple2. We found that CDK1 phosphorylation targets were enriched in the TF-PPI network that regulates the expression of Mediumpurple2 genes. In addition, pharmacological and genetic perturbations of *CDK1* altered the expression of Mediumpurple2 genes in human cancer cell lines, and the expression levels of *CDK1* and Mediumpurple2 genes were correlated under PD-like insult and levodopa treatment. However, regulation of Mediumpurple2 gene expression by CDK1 is complex, as knockdown and overexpression of *CDK1* both elevated the expression of Mediumpurple2 genes in human cancer cell lines, and the direction of correlation between *CDK1* and Mediumpurple2 gene expression in striatal MSNs appeared to be cell-type specific. Given the large size of the Mediumpurple2-regulating TF-PPI network, it is possible that this complex modulation by CDK1 is associated with the exact molecular cascades that were disrupted or activated by different CDK1 perturbations. In addition, CDK1 might be only one of many potential regulators upstream of this large TF-PPI network, as suggested by the kinase enrichment analysis. While these complications require further studies to understand the exact nature of CDK1 regulation of the Mediumpurple2, our observations may also be related to the complex cellular functions of CDK1.

Aside from their function as key mitotic regulators, reactivation of CDKs in post-mitotic neurons have been linked to neuronal death in multiple neurodegenerative disorders, including PD^58,59^. However, the striatum is not the primary region of neurodegeneration in PD, and we found the Mediumpurple2 network was likely to function downstream of the dopamine receptors in striatal neurons. Therefore, altered gene expression in this *CDK1*-regulated mitotic gene network may not be related to signaling pathways underlying neuronal death but instead involved in the postsynaptic regulation of sleep and affective functions in response to the decreased dopamine levels in PD, eventually leading to PD symptoms. Interestingly, a number of mitotic genes, including those regulated by CDK1, indeed function beyond cell cycle or neuronal death and are involved in postsynaptic regulation of synaptic plasticity^60^. Administration of flavopiridol, a non-specific CDK inhibitor, alleviates MPTP-induced Parkinsonian motor symptoms without restoring striatal dopamine levels^61^, suggesting the possibility of a postsynaptic function of CDKs in striatal dopamine signaling. While the role of CDK1 and mitotic genes in postsynaptic dopamine signaling has not been experimentally demonstrated, a non-mitotic CDK, CDK5, has been shown to suppress dopamine D1 signaling in the striatum by phosphorylation of postsynaptic protein DARPP-32^62^. Given our finding that the CDK1-regulated dopamine-responsive network was strongly associated with sleep and affective functions that were prone to disruption in PD, it is tempting to hypothesize that the postsynaptic function of CDK1 involves regulation of sleep and affective functions, which is disturbed in PD, leading to sleep and mood symptoms. In line with this hypothesis, a role of CDK1 in sleep regulation has recently been demonstrated in a group of newly identified sleep/wake-controlling neurons in the *Drosophila* brain^63,64^.

One of the substrate proteins phosphorylated by CDK1 and an upstream TF of the Mediumpurple2 network, NUCKS1, links this CDK1-regulated dopamine-responsive network to the genetic susceptibility of PD, further demonstrating the importance of Mediumpurple2 network in PD. *NUCKS1* has been reported as one of the candidate genes of the *PARK16* locus in multiple genetic association studies^12,65–68^, although the potential mechanisms by which the *PARK16* locus influences the risk of PD is unknown. Our analysis found that the *NUCKS1* eQTL and a PD meta-GWAS locus are colocalized, suggesting that PD genetic risk influences the striatal expression of *NUCKS1*. Since transcriptional targets of NUCKS1 were enriched in the Mediumpurple2 network, our finding raises an intriguing hypothesis that genetic variations at the *PARK16* locus contribute to the risks of PD at least in part by influencing the expression of *NUCKS1*, which in turn regulates a striatal gene network sensitive to dopamine and important for PD symptoms.

In summary, our work provides a framework for utilizing multiple types of datasets in mice and humans to provide insights and generate hypothesis regarding how disease pathology impinges on diverse biological functions and leads to a complex spectrum of symptoms. Results from our integrated systems analysis highlighted a dopamine-modulated network, Mediumpurple2, which was disrupted in PD and links co-regulated expression of a number of cellular pathways, including mitosis, circadian clock regulated gene expression, and Notch signaling, to sleep fragmentation, despair-related phenotypes, as well as motor functions. These results implicate that disrupted network function in PD may be key to the spectrum of motor, sleep and mood symptoms. Our unbiased approach also found CDK1 as a candidate regulator of the network, as suggested by multiple analyses. Although each of these analyses alone may only provide an indirect implication, together, they consistently support a link between CDK1 and gene expression of the Mediumpurple2 network. Taken together, our analyses suggested candidate gene network and its potential regulator for future detailed studies to test the molecular cascades important for the emergence of PD non-motor symptoms. Future efforts as such may ultimately shed light on novel therapeutic strategies that treat PD by restoring the normal expression pattern and the functionality of gene networks downstream of dopamine signaling.

## Methods

### Meta-analysis of differential gene expression in PD

A total of five striatal transcriptomic datasets were identified (GSE28894, GSE23290, GSE54282, GSE20291, and Miller *et al*., 2006 supplementary data; for details see Supplementary Table S1)^69–72^. As different microarray platforms were used and raw data was not available in one of the datasets, a uniform processing and normalization of raw microarray data were not possible. We thus downloaded the quantile-normalized probeset-level data (four datasets from the GEO database and one directly from supplementary data of the publication). Log2-transformation was applied as needed. In order to control for the effects of known and hidden covariates in each of the datasets, we used R/sva package to first adjust expression data for known factor covariates (such as sex) with the combat function and then estimate surrogates for hidden covariates with the sva function. Known numeric covariates (such as age and postmortem interval, when available) and estimated surrogates were then evaluated by fitting the expression data a linear model and comparing the distribution of model P values against uniform distribution, in order to select covariates with widespread effects. Selected covariates were further adjusted for by fitting the expression data with a robust linear model and taking intercept + residuals as the adjusted expression values. Within each dataset, the expression values were further standardized to μ = 0 and σ = 1, and effect sizes (Hedges’ g) of disease status were then computed for each gene. We condensed the expression profile to the gene level, keeping the probeset with the largest effect size (i.e., the smallest dataset-specific P value and thus the least likely by chance) to represent the gene^22^. 10,098 genes were included in all datasets and thus were used for meta-analysis. We used the R/GeneMeta package to combine effect sizes across datasets with a random effect model, compute meta-Z-score statistics, and estimate false discovery rate (FDR) by 1000 permutations.

### Differential gene expression signatures in mouse models of PD

We found 9 datasets (GSE52584, GSE60413, GSE60414, GSE20547, GSE8030, GSE31458, GSE60080, and GSE7707) from 7 studies profiling gene expression in the striatum of mouse models of PD^73–78^. We also downloaded data (GSE55096) from a study profiling gene expression in the *Drd1*+ and *Drd2*+ neurons in a mouse 6-OHDA model of PD with chronic levodopa treatment^30^. All these studies used Affymetrix arrays (N = 3–12 per group per dataset), and the RMA-normalized and log2-transformed expression values downloaded from GEO were used for differential expression analysis. Data were condensed to gene-level and analyzed in the same manner as in the meta-analysis of human PD datasets, except that dataset-specific Z-scores (rather than a combined meta-Z-score) were computed for each mouse model of PD at a particular age or in a specific type of neurons.

### Mouse striatal gene networks

Details of the chronically stressed (B57CL6/J x A/J) F2 mice have been described in our previous publication^19^. Briefly, male F2 mice (N = 338) at 4–5 weeks of age were ordered from the Jackson Laboratory (Bar Harbor, ME). All mice were individually housed in opaque cages to create social isolation and were then subjected to a battery of stressors, including novel exposed environments, restraint, forced swimming, fear conditioning, social defeat, cold exposure, a 6-hr fast followed by glucose challenge, and 6-h sleep deprivation, and a second episode of restraint during the time of sleep recordings. A comprehensive set of phenotypes were measured during the stress treatment. In addition, after the fast and glucose challenge, mice were surgically implanted with EEG and EMG electrodes for sleep recordings, which included a 24-h recording of undisturbed bassline sleep/wake, a 24-h recording of recovery after sleep deprivation, and a 24-h recording of recovery after restraint stress. Behavioral and sleep phenotypes were grouped into categories according to prior knowledge and factor analysis. After sleep recordings, animals were left undisturbed for 2 weeks before euthanasia and tissue collection. RNA was isolated from the striatum of a subset of randomly selected 100 animals, and RNA-sequencing for gene expression profiling was performed using the Illumina HiSeq 2500 System with 100 nucleotide single-end reads. Sequencing reads were mapped to Ensembl NCBIM37 mouse reference genome using TopHat, and raw count values were deduced using HTSeq. Data has been deposited to the GEO database (GSE60312). Coexpression networks and modules were reconstructed using weighted gene coexpression network analysis (WGCNA), and module-phenotype relationships were determined by correlating module eigengenes to phenotype values. 1000 sample permutations were used to estimate the FDR. For each phenotypic category, the relevance of a module was determined by ranking modules according to the number of significant correlations with the phenotypes of the category.

### Enrichment tests

Gene set enrichment analysis (GSEA)^24^ was used for testing enrichment between a gene set (e.g., mouse coexpression network modules or gene functional groups) and a differential gene expression signature (e.g., PD meta-signature, signatures of PD mouse models, or signatures of CDK1 perturbations). Briefly, the gene-level differential expression statistics were ranked-ordered, and a Kolmogorov-Smirnov-like enrichment score was computed to evaluate whether the gene set was enriched at one end of the ranked profile. The labeling of gene names in the differential expression signature was permuted 10,000 times to estimate P values. For testing enrichment between two gene sets, (for example, the enrichment of mouse module genes in gene functional pathways or cell-type signatures), Fisher’s exact test was used. P values from GSEA or Fisher’s exact test were adjusted for multiple testing using either Bonferroni correction or FDR as appropriate. Gene sets of cellular functions and pathways from multiple databases were downloaded from MSigDB and Enrichr^24,79^ (accessed on 3/22/2017).

### Drug signature analysis

To calculate drug signature enrichment, transcriptomic data of 6,100 individual experiments from the Connectivity Map^28^ were merged into representative signatures for each of the 1,309 unique small molecule compounds by computing the prototype ranked list^80^. To quantify the tendency for a compound to up or downregulate a gene network, a modified Kolmogorov-Smirnov score was calculated^28^. Drug signatures were permutated 1000 times to derive an empirical null distribution of the score for computing P values and estimating FDR (Benjamini-Hochberg). To investigate target enrichment, we used a chemogenomic enrichment method and computed a Kolmogorov-Smirnov-like score to evaluate the over or under-representation of drug-induced transcriptional profiles with ancillary annotation resources of drug classes and known/predicted targets^31^.

### Expression relationships between *CDK1* and Mediumpurple2 network

We downloaded from GEO (GSE31534 and GSE31912) the RMA-normalized and log2-transformed microarray data of an RNAi screen study in human cell lines^33^. Differential expression analysis was performed using the R/limma package. GSEA was then used to test whether the Mediumpurple2 (homologous genes in humans) was enriched in the differential expression signature.

We also utilized data generated by the NIH LINCS program^34^, which uses a measured set of ~1000 genes to computationally infer genome-wide differential gene expression in response to various perturbations in 9 human cancer cells (i.e., the L1000 data). We downloaded from GEO (GSE92742) the gene-level modZ scores (i.e., the aggregated Z score from replicates in the same cell line) computed from experiments of CDK1 RNAi or overexpression in each cell line. We derived a consensus CDK1 RNAi or overexpression signature across all cell lines by summing the modZ scores to a single Z score for each gene using the Stouffer’s Z method. The summed Z scores were then used in GSEA as the differential expression signature of CDK1 RNAi or overexpression to test the enrichment of human homologs of Mediumpurple2 genes.

To test the correlation between CDK1 expression and Mediumpurple2 in striatal *Drd1*+ and *Drd2*+ MSNs, we used data (GSE55096) of gene expression profiling in a mouse 6-OHDA model of PD chronically treated with levodopa with a high or low dose^30^. We computed Pearson’s r between the expression of *CDK1* and all other genes across the genome, and transformed the Pearson’s r to a Z score using Fisher z-transformation. Z scores were then used in GSEA to test whether the Mediumpurple2 genes were enriched with those most strongly correlated with CDK1.

### TF-PPI network

ChIP-on-chip- or ChIP-seq-determined sets of TF targets genes from ChEA^36^ and ENCODE^37^ were downloaded from Enrichr^79^ (accessed on 3/22/2017). P values were computed from Fisher’s exact test to evaluate the enrichment of the Mediumpurple2 genes in each of the TF target gene set. We combined the P values from multiple target gene sets (e.g. different cell lines or conditions) of the same TF to a single P value for each of the unique TF using Fisher’s combined probability test. FDR was estimated using the Benjamini-Hochberg procedure. For significant TFs (FDR < 0.01), we constructed a protein interaction network to include their immediate binding partners, using experimentally determined PPI data in mice and humans curated by the BioGRID database^81^ (v3.4.153, accessed on 10/28/2017). The combined TF-PPI network was then trimmed to remove low-expression genes in the striatum according to the (C57BL/6J x A/J) F2 RNA-seq data, in order to reflect high-confidence transcriptional regulation of the Mediumpurple2 module that is likely to occur in the striatum. Using cell-type signatures characterized by TRAP^29^, we validated that the trimmed TF-PPI network of Mediumpurple2, like the Mediumpurple2 gene coexpression network itself, was enriched specifically with signatures of striatal *Drd1*+ and *Drd2*+ neurons but not cholinergic neurons (Supplementary Table S2).

### PD GWAS-eQTL colocalization test

We downloaded the summary statistics of the top 10,000 significant PD-associated SNPs from the PDgene database^41^ (assessed 11/19/2015) and summary statistics of eQTL results in the caudate and the putamen from the GTEx^42^ data portal (assessed 4/27/2017). Colocalization test was performed using the R/coloc package, which uses a Bayes test to evaluate posterior probabilities for colocalization of genetic associations from summary statistics^43^. A posterior probability of 0.75 or larger was used to call colocalization between PD GWAS loci and striatal eQTL.

## Supplementary information

Supplementary information

Table S1

Table S2

## Data Availability

All data generated or analyzed during this study are included in this published article (and its Supplementary Information files).
