# Supplementary material for: Parkinson’s Disease is Associated with Dysregulations of a Dopamine-Modulated Gene Network Relevant to Sleep and Affective Neurobehaviors in the Striatum
Source: Sci Rep. 2019 Mar 18;9:4808. doi: 10.1038/s41598-019-41248-4 (PMC6423036; doi:10.1038/s41598-019-41248-4)
Supplement: Supplementary file 1 — Supplementary information [file 41598_2019_41248_MOESM1_ESM.docx]

**SUPPLEMENTARY INFORMATION**

**Parkinson’s Disease Is Associated with Dysregulations of a Dopamine-Modulated Gene Network Relevant to Sleep and Affective Neurobehaviors in the Striatum**

Peng Jiang^1^, Joseph R. Scarpa^2^, Vance D. Gao^1^, Martha H. Vitaterna^1^, Andrew Kasarskis^2^, Fred W. Turek^1, 3^*

^1^ Center for Sleep & Circadian Biology, Department of Neurobiology, Northwestern University, Evanston, IL 60208, USA.

^2^ Icahn Institute for Genomics and Multiscale Biology, Department of Genetics and Genomic Sciences, Icahn School of Medicine at Mount Sinai, New York, NY 10029, USA.

^3^ Department of Neurology, Northwestern University Feinberg School of Medicine, Chicago, IL 60611, USA.

Correspondence: Fred W. Turek, PhD ([fturek@northwestern.edu](mailto:fturek@northwestern.edu))

**SUPPLEMENTARY FIGURES**

**
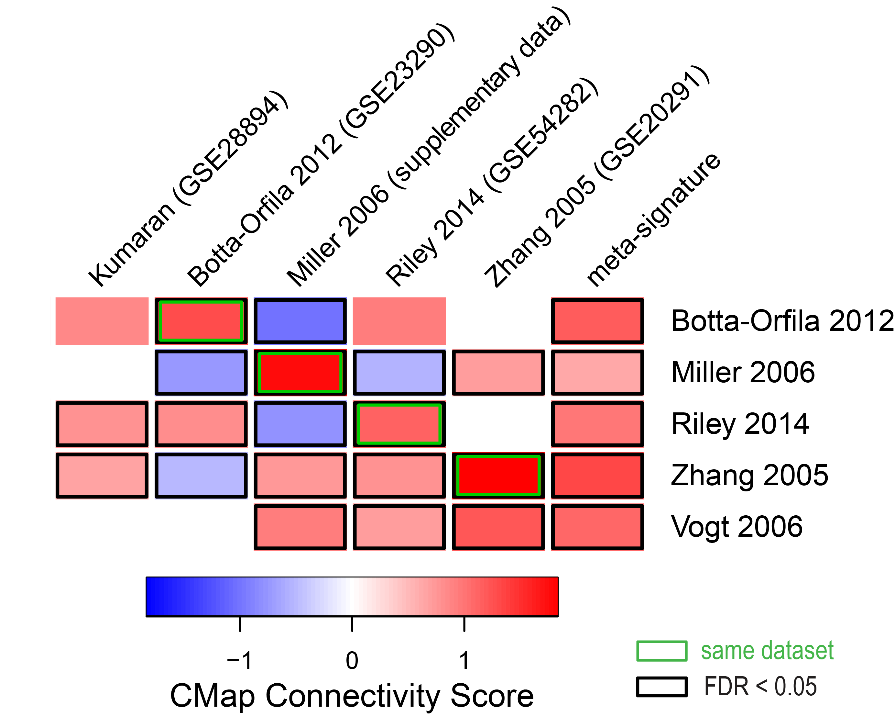
**

**Figure S1.** **Heatmap of connectivity scores comparing differential expression signatures from our re-analysis of individual datasets or the meta-analysis (columns) and previously reported sets of differentially expressed genes in PD (rows).** For dataset and gene set information, see **Supplementary Table S1**. Positive connectivity scores indicate differentially expression signatures and reported genes are matched in the same direction, and negative scores indicate matching in the opposite direction. Highest positive scores were observed between the signatures from our re-analysis of individual datasets and the reported gene sets from studies using the same datasets (green rectangles), indicating good consistency between our re-analysis and reported results. Heterogeneity among study cohorts and datasets as also observed, as the connectivity scores were highly variable and were not always positive when comparing our re-analyzed signatures to the reported gene sets from different datasets. Despite the heterogeneity among individual datasets, the meta-signature showed strong positive connectivity to the reported genes sets from all the datasets that were included in the meta-analysis. The meta-signature was even positively connected to the differentially expressed genes reported in a study (i.e., Vogt 2006) that was not included in the meta-analysis due to unavailability of expression data, suggesting that the meta-signature robustly captured striatal transcriptomic alterations across PD cohorts.

**SUPPLEMENTARY METHODS**

**The connectivity score**

The connectivity score was developed by the Connectivity Map (CMap) project to measure alike (i.e., positive connectivity) or opposite matchings (i.e., negative connectivity) between a signature (i.e., a rank-ordered list of gene-level differential expression statistics across the genome) and a set of differentially expressed genes selected at a threshold^26^. Briefly, two separate Kolmogorov-Smirnov-like enrichment scores (i.e., *KS_up_* and *KS_down_*) were used to evaluate the enrichment of up-regulated and down-regulated genes, respectively, at the one end of the differential expression signature. The connectivity score of between the gene set and DE signature was set to 0 if *KS_up_* and *KS_down_* have the same sign, and was otherwise set to *KS_up_ - KS_up_.* Unlike the original report^26^, the connectivity score used in this study was not normalized to the max of the absolute values of connectivity scores from all gene sets tested, allowing for comparison across sets of differential expression signatures. FDR was estimated by permuting the gene labels of the differential expression signature 1000 times.

**SUPPLEMENTARY TABLE CAPTIONS**

**Supplementary Table S1**: Meta-analysis of differential gene expression in the striatum of PD patients. Summary of datasets used in the analysis, meta-differential analysis results, pathway enrichment results, and previously reported gene sets are given in tabs.

**Supplementary Table S2:** Transcription factors whose targets were enriched in the Mediumpurple2 module and the transcription factor protein-protein interaction network. Tabs contain the TF enrichment statistics, pairs of interacting proteins in the TF PPI network, and the cell-type signature enrichment statistics for the TF PPI network.
